# MicroRNA-21-5p profile in the alveolar bone following tooth extraction in medication-related osteonecrosis of the jaw rat model

**DOI:** 10.3389/fdmed.2024.1477274

**Published:** 2024-12-17

**Authors:** Manta Morakotsriwan, Theerapat Chanamuangkon, Anjalee Vacharaksa, Pirawish Limlawan

**Affiliations:** ^1^ Faculty of Dentistry, Chulalongkorn University, Bangkok, Thailand; ^2^ Biomaterial Testing Center, Faculty of Dentistry, Chulalongkorn University, Bangkok, Thailand; ^3^ Department of Microbiology, Faculty of Dentistry, Chulalongkorn University, Bangkok, Thailand; ^4^ Department of Oral Medicine, Faculty of Dentistry, Chulalongkorn University, Bangkok, Thailand; ^5^ Center of Excellence and Innovation for Oral Health and Healthy Longevity, Faculty of Dentistry, Chulalongkorn University, Bangkok, Thailand

**Keywords:** alveolar bone, bisphosphonates, bone remodeling, bisphosphonate-associated osteonecrosis of the jaw, MicroRNA-21

## Abstract

**Objective:**

This study aimed to compare microRNA-21-5p expressions at the extraction wound in the maxillary bones of rats with medication-related osteonecrosis of the jaw (MRONJ) and normal rats at different time points.

**Materials and methods:**

In total, 18 female, 8-week-old Sprague-Dawley rats were randomly assigned to the experimental group (*n* = 9) and the control group (*n* = 9). To establish MRONJ in the right maxillary first molar area in the experimental group, zoledronate (66 µg/kg) and dexamethasone (5 mg/kg) were administered intraperitoneally every other day for 2 weeks before tooth extraction. Normal saline was administered in the control group. After tooth extraction, the drugs were continuously administered until the experimental endpoints, namely 1, 14, and 28 days post-tooth extraction. At each endpoint, three rats from each group were euthanized. The maxilla bones at the wound area were harvested. A real-time polymerase chain reaction (RT-PCR) was performed to compare the expression levels of miRNA-21-5p at each time point between the MRONJ group and the control group.

**Results:**

From their gross appearance, the rats that received zoledronate and dexamethasone developed MRONJ as demonstrated by non-healing wounds and exposed bone at 14 and 28 days post-extraction in contrast to the controls. The RT-PCR showed that the expression levels of miRNA-21-5p were relatively higher in the MRONJ rats compared to the control rats at day 14 and then the difference was lower at day 28 post-tooth extraction.

**Conclusion:**

The findings indicated that the microRNA-21-5p expression levels varied during the socket healing process in the MRONJ rats, reaching a peak at 2 weeks after tooth extraction.

## Introduction

Osteoporosis, associated with various factors such as menopause and aging, is the most prevalent chronic metabolic bone disorder, characterized by heightened bone fragility due to lower bone volume. While it occurs across all age groups and in both sexes, it is more prevalent among the elderly and females. Due to an older population and extended life expectancy, osteoporosis is progressively emerging as a global epidemic (1).

Antiresorptive medication is extensively utilized for its efficacy in diminishing the chance of bone fractures in osteoporosis and bone malignancy. Bisphosphonates (BPs) are the most used group of antiresorptive drugs (2). BPs are derivatives of inorganic pyrophosphate and are capable of binding to hydroxyapatite crystals, resulting in the inhibition of hydroxyapatite breakdown; this allows BPs to suppress bone resorption (3). Research has also demonstrated that BPs stimulate bone formation by increasing osteoblast proliferation and preventing both osteoblast and osteocyte apoptosis (4). BPs also suppress bone resorption by promoting osteoclast apoptosis (5) and upregulating osteoprotegerin (OPG), the competitor of the receptor activator of nuclear factor kappa-B ligand (RANKL), the osteoclastogenesis promoter (6).

Patients with menopausal osteoporosis commonly receive injections of zoledronic acid, a member of the BP group (7). However, the adverse effects of BP medication have also been reported in the jaw area and are known as medication-related osteonecrosis of the jaw (MRONJ), which can severely impact the quality of life of patients (8, 9). The symptoms of MRONJ vary and include delayed healing post-extraction, halitosis, severe pain, difficulty when chewing, and dysphagia. Studies have shown that the incidence of medication-related osteonecrosis of the jaw in patients who were previously exposed to intravenous bisphosphonates varies from 1.6%–14.8% (10). Moreover, a significant difference in MRONJ development was confirmed with the use of injections compared with oral medication administration (odds ratio = 5.01) (11). Several protocols have been introduced for the management of patients with MRONJ, both surgically (12) and conservatively (13). However, a consensus on the effective gold standard for treatment has not yet been established.

Recently, researchers have found bioactive molecules that play crucial roles in controlling diverse physiological cellular and metabolic pathways by regulating gene expression which are called small non-coding RNAs (sncRNAs). sncRNAs are RNAs with less than 200 nucleotides and are usually non-coding (14). MicroRNAs (miRNAs) are one of several types of sncRNAs. MiRNAs are small evolutionarily conserved single-stranded non-coding RNA molecules transcribed from DNA. MiRNAs act as post-transcription regulators in the cytoplasm by base-pairing with the untranslated regions of the target mRNA. The level of complementarity between the miRNAs and target mRNA determines which silencing mechanism is employed, i.e., cleavage and degradation or translation inhibition (15).

MiRNAs modulate bone formation (16) and resorption (17), therefore facilitating the preservation of bone homeostasis. In pathological settings, abnormal miRNA signaling contributes to the initiation and advancement of skeletal diseases, including osteoporosis (18). Moreover, miRNAs can be released into circulation in extracellular vesicles and have therapeutic promise as a non-invasive biomarker. In a therapeutic context, the administration or antagonism of miRNA has been shown to influence several diseases in pre-clinical settings, thereby emerging as a promising therapeutic strategy (19).

MiRNA-21 was shown to be involved in the early bone remodeling process of tooth extraction wound healing in mice as the bone percentage in the alveolar socket of miRNA-21-deficient mice was lower than in wild-type mice. Yet, the biological pathway behind this phenomenon was not investigated (20). However, another study demonstrated that miRNA-21 promotes the migration and osteogenic differentiation of bone marrow-derived stem cells (BMSCs). The osteogenic ability of BMSCs is promoted by increasing P-Akt and HIF-1*α* activation in the PTEN/PI3K/Akt/HIF-1*α* pathway (21). MiRNA-21 was also involved in orthodontic tooth movement bone remodeling as osteoclast number, RANKL expression, RANKL/OPG ratio, and alveolar osteoclastogenesis were decreased in miRNA-21-deficient mice compared to wild-type mice. The decreased tooth movement was due to the lower bone resorption in the miRNA-21-deficient mice (17, 22).

However, the involvement of miRNA-21 in MRONJ has not yet been studied in detail even though miRNA-21 has been reported to promote osteogenic differentiation (21), bone remodeling, and osteoclastogenesis (17). To the best of our knowledge, there is no research regarding miRNA-21 expression in the affected bone area. Studying the miRNA-21 profile in the MRONJ lesion could be beneficial as the results may be used to provide a promising candidate for miRNA therapy in patients who are administered bisphosphonates and develop MRONJ.

## Materials and methods

### Animal procedures

All the procedures were approved by the Chulalongkorn University Laboratory Animal Center (protocol number 2273009) before any experiments. The MRONJ rat models were created according to previously published literature (23).

We based the number of animals on parameters from related published articles that achieved meaningful data in animals. The mean and standard deviation are based on the occurrence of osteonecrosis in two experiment groups after receiving bisphosphonate and tooth extraction in the study by Barba-Recreo et al. (24). The required number animals was calculated using a power analysis in G*power software to provide a statistical power of 0.8 and type I error of 0.05. The total number/group that was calculated from this formulation was three rats per group. Therefore, 18 Sprague-Dawley rats were used in the study. Thus, 7-week-old female healthy rats with body weights of approximately 150–200 g were delivered to the animal facility and quarantined for 1 week before the experiments.

MRONJ models preparation: Before tooth extraction, all rats were randomly assigned to one of two groups by a simple randomized method as follows:

Group 1 (Experiment) (*n* = 3/timepoint): administration of zoledronate (66 µg/kg) and dexamethasone (5 mg/kg)
Group 2 (Control) (*n* = 3/timepoint): administration of normal saline solution

A total of 18 rats were used, with three samples in each group at three time points.

To begin each experiment, the drugs, as specified above, or normal saline solution, were injected intraperitoneally into the rats in each group. Drug administrations were repeated every other day and continued for 4 weeks. After drug administration, the rats were monitored and weighed every 2–3 days (24).

Two weeks after the drug administration (day 0), all rats had their maxillary first molar extracted. As a pre-operative analgesic, carprofen (Rimadyl®, Zoetis USA) was injected subcutaneously (5 mg/kg) to provide moderate pain relief. Enrofloxacin (Baytril®, Elanco USA) was also injected subcutaneously (5 mg/kg) as a prophylactic antibiotic. General anesthesia was conducted using an intraperitoneal injection of tiletamine-zolazepam (Zoletil®; 20 mg/kg) and xylazine (2 mg/kg). After general anesthesia was reached, it was confirmed by tail pinching and corneal reflex. Local anesthesia was then conducted with 2% mepivacaine and 1:100,000 epinephrine (Scandonest®, Septodont USA). Tooth extraction was done by first locally infiltrating the buccal mucosa of the maxillary right first molars, and then extracting the tooth. The rats were monitored until they were fully recovered from the general anesthesia. For postoperative care, the animals were transferred to a heating pad to recover from the anesthesia. The animals were monitored continuously for 2 h to observe any signs of labored respiration and then transferred to a cage with free access to food and water after showing signs of responsiveness. Acetaminophen (in the drinking water) was provided for 7 days after the surgery (23).

After the tooth extraction, the rats were observed and weighed every day for 7 days to ensure normal eating behavior and nutrition. A soft diet was provided for all the rats after tooth extraction for 7 days and this was changed into a normal diet 7 days post-tooth extraction. On days 1, 14, and 28 post-tooth extraction, three rats from each group (a total of six rats per time point) were euthanized by inhalation of CO_2_. Confirmation of death was done by cervical dislocation before the maxillae were harvested. The gross wound healing of the extraction sockets on the maxillae was photographed.

After being photographed, the alveolar bone of the extraction wound area was excised into cubes with the size of approximately 2 × 2 × 2 mm^3^ and collected in tubes containing RNAlater solution (Thermo Fisher Scientific, Cat. no: AM7020) for RNA extraction and quantitative polymerase chain reaction (qPCR) analysis of miRNA-21-5p expression levels.

### RNA isolation, reverse transcription, and quantitative PCR

The collected bone tissues were placed in a ceramic bead tube (PowerBead tube, Qiagen, Cat. no: 13113-50) and homogenized using a PowerLyzer 24 Homogenizer (Qiagen) at 4,200 Hz for 45 s. A PAXgene® Tissue RNA/miRNA Kit (Qiagen, Cat. no: 766134) was used for miRNA isolation and purification according to the manufacturer’s protocol.

After RNA quantification using a NanoDrop 2000 (Thermo Fisher Scientific, Cat. no.: 3377156), 400 µg of total RNA was used for reverse transcription using miRCURY LNA RT Kit (Qiagen, Cat. no: 339340) in a thermocycler (CFX 96 Touch, Bio-Rad, Hercules, USA).

The quantitative PCR was then performed using a miRCURY LNA miRNA SYBR® Green PCR kit (Qiagen, Cat. no: 339345) in a PCR detection system (CFX 96 Touch, Bio-Rad, Hercules, USA). The primers of miRNA-21-5p and snRNA of the reference gene U6 were ordered from Qiagen (miRCURY LNA miRNA PCR Assays, Cat. no: 339306). The PCR conditions were 95°C for 2 min followed by 40 cycles of amplification consisting of 95°C for 10 s and 56°C for 60 s as per the manufacturer's protocol. All the samples were run in duplicate and the results were averaged for the gene expression analysis. The expression level of miRNA-21-5p was normalized to reference gene U6. The fold expression of miRNA-21 compared to the untreated controls was calculated using the 2^–ΔΔC^ method.

### Statistical analysis

The data were expressed as the mean values ± standard deviation. Data normality was tested, and statistical analyses were performed using one-way analysis of variance (ANOVA) and Tukey’s *post-hoc* test in the SPSS v21.0 statistical software package. The differences were considered statistically significant when the *p*-value was ≤0.05. The graph illustration was created using Microsoft Excel and PowerPoint software.

## Results

### Gross characteristics

**Figure 1:**
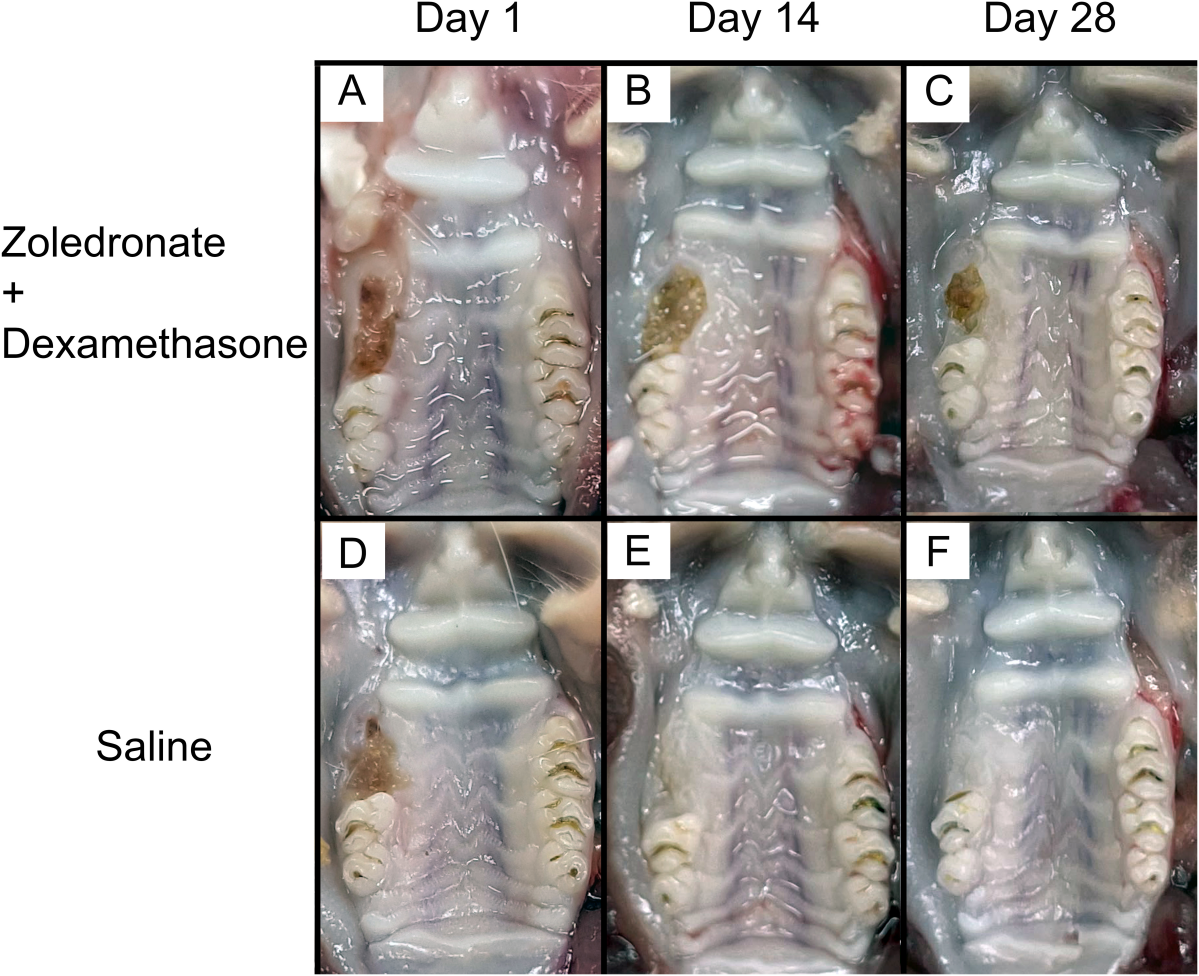
Gross characteristics of the right maxillary molar extraction sockets of **(A)** an MRONJ model, day 1 post-extraction; **(B)** an MRONJ model, day 14 post-extraction; **(C)** an MRONJ model, day 28 post-extraction; **(D)** a control, day 1 post-extraction; **(E)** a control, day 14 post-extraction; **(F)** a control, day 28 post-extraction.

The gross appearance of the maxillae in the rats that received zoledronate and dexamethasone showed unhealed extraction sockets at all time points. However, in the control group that received saline alone, the wounds were almost completely healed on day 14 post-extraction and then completely healed on day 28 post-extraction. This indicated that MRONJ occurred in the experimental group (Figure 1).

### MiRNA-21-5p expression level

**Figure 2:**
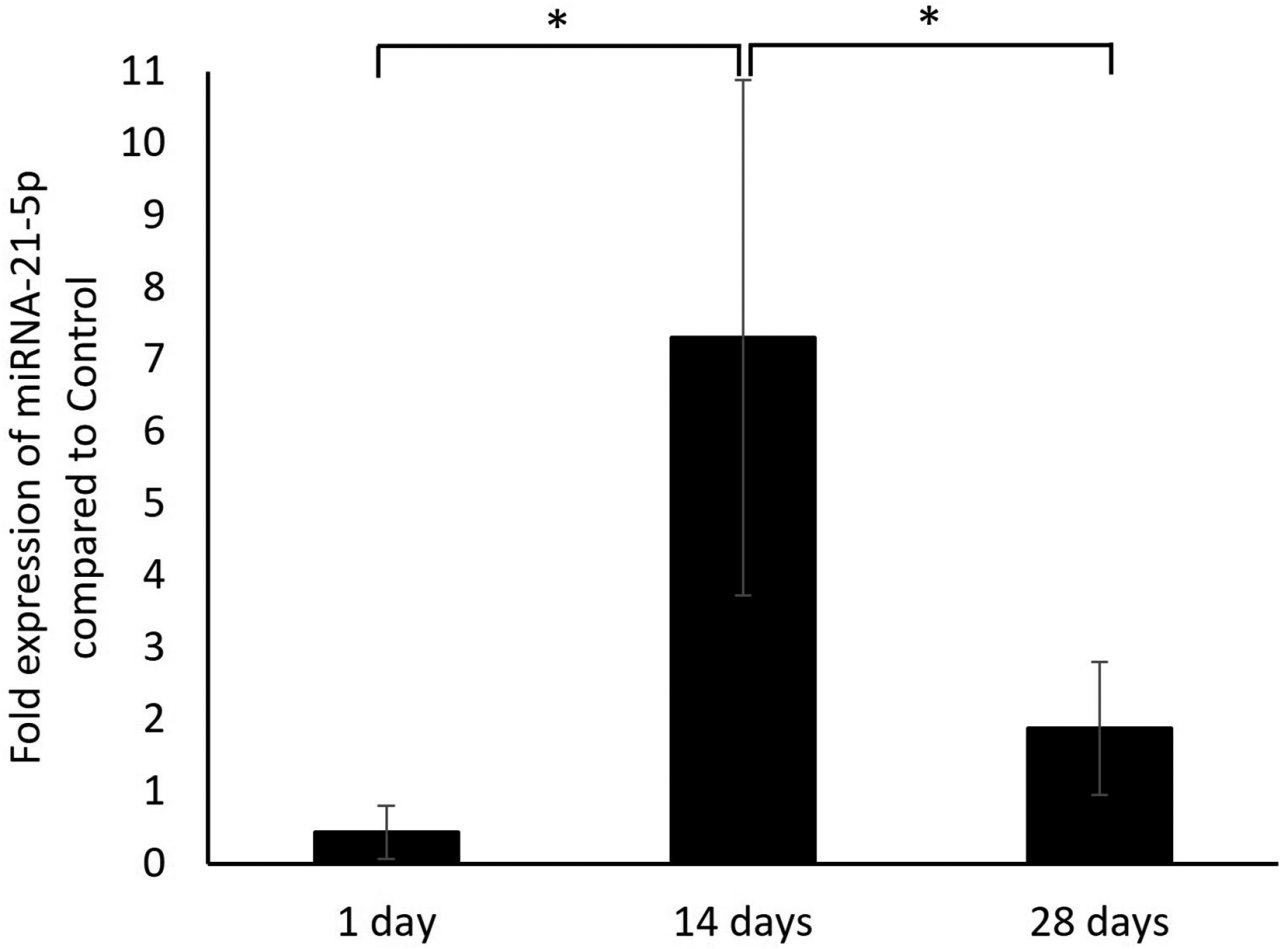
The fold expression level of miRNA-21-5p in the alveolar bone of extraction wound on days 1, 14, and 28 post-extraction in the rats that received zoledronate and dexamethasone compared to the controls. *p < 0.05.

In the qPCR analysis (Figure 2), the expression level of miRNA-21-5p in the alveolar bone extraction wounds increased from day 1 to day 14 after tooth extraction as there was a significantly higher expression of miRNA-21-5p on day 14 (7.3 ± 3.58) compared to day 1 (0.43 ± 0.37). From the results, miRNA-21-5p expression level peaked at 2 weeks post-extraction. The miRNA-21-5p expression level then decreased as on day 28 there was a significantly lower expression (1.88 ± 0.92) compared to day 14. Even though the expression of miRNA-21 on day 28 was higher than on day 1, there was no statistical difference.

## Discussion

MRONJ is defined as persisting exposed bone or bone that can be probed through the fistula in the maxillofacial region for more than 8 weeks in patients who received antiresorptive drugs without a history of radiation therapy (10). In our experiments, the rats that received zoledronate and dexamethasone demonstrated non-healing wounds and exposed bone 4 weeks after maxillary first molar extraction. This suggested that the rats developed MRONJ, as our previous work showed histologically necrotic bone at 4 weeks in the unhealed extraction wounds of rats that received zoledronate and dexamethasone (23). Several studies using rat models also found the occurrence of MRONJ at this time point (25, 26). In this study, injections of a combination of bisphosphonate and dexamethasone to induce MRONJ in a rat model were administered, consistent with the previous studies (27, 28), to simulate the clinical situation in which patients with multiple myeloma routinely receive bisphosphonate and dexamethasone as part of their treatment. Furthermore, for solid tumors, the patient not only undergoes surgical removal (29) but also receives corticosteroids in association with chemotherapy (30).

According to the Scottish Dental Clinical Effectiveness Programme’s (SDCEP) Oral Health Management of Patients at Risk of Medication-related Osteonecrosis of the Jaw guidelines, patients receiving antiresorptive agents are categorized into different levels of risk of MRONJ. The factors include their medical condition and other medications they are receiving. For example, patients receiving glucocorticoids along with BPs have an increased risk of MRONJ (31). This situation was imitated in this study.

In this study, all the animals were controlled to be of the same sex to maintain similarities between each animal. Females were selected to mimic the clinical situation where most patients who receive zoledronic acid injections are women with menopausal osteoporosis (7). The age of 8 weeks was selected instead of an older age to represent a realistic scenario, as the challenges associated with tooth extraction in older rats may elevate the risk of mortality following the procedure.

MiRNAs are post-transcriptional gene regulators involved in many physiological and pathological pathways. Several research studies have been conducted to discover the role of miRNA in the pathogenesis of MRONJ, as reviewed by Mohd Yunus et al. (32). In patients with multiple myeloma who received bisphosphonates, the expression levels of several miRNAs were significantly higher in the peripheral lymphoid compartment of those who developed MRONJ compared to those who did not. The miRNAs included miR-16-1, miR-21, miR-23a, miR-28, miR-101-1, miR-124-1, miR-129, miR-139, miR-145, miR-149, miR-202, miR-221, miR-424, and miR-520 (33). For miRNA-21, the research study in search of a diagnostic biomarker quantified the miR-21 expression level in the serum of an MRONJ rat model and found that it was upregulated during disease progression as there was a higher expression of the miRNA in the serum of the MRONJ rats compared to the controls at 4 and 8 weeks but not at 1 week (34). In our experiment, we investigated the expression level of miRNA-21-5p in the affected alveolar bone and found a higher expression at 2 weeks but not at 4 weeks. This could be due to the different areas of sample collection, as the expression levels of the same miRNA have been found to be different between the tissue and serum in cancer patients (35). Another study on colorectal cancer reported no overlap in the miRNAs expressed in the tissue and serum and suggested that serum-regulated miRNAs may not be actively secreted from the cancer cells (36).

In this study, the expression levels of miRNA-21-5p in the MRONJ lesions reached their peak at 2 weeks after tooth extraction, which could be due to the need for miRNA-21 in several healing-associated pathways at this time point. The variation in miRNA-21-5p expression among samples within the same group may be due to differences in wound severity among the rats. Despite the operator being the same, the dimensions of the wound are unlikely to be equivalent; hence, the requirement for miRNA-21-5p on day 14 varied. However, on day 28, the standard deviation decreased, possibly due to the diminished requirement for miRNA-21-5p, resulting in reduced expression and thus a smaller deviation across the samples.

The pathophysiology of MRONJ has been hypothesized to be bone remodeling inhibition, inflammation or infection, and angiogenesis inhibition (10) and miRNA-21 has been reported to be involved in these pathways. Regarding osteogenesis, miR-21 promotes osteogenic differentiation of bone marrow mesenchymal stem cells by targeting inhibitory Smad7 in the Smad7-Smad1/5/8-RUNX2 pathway (37). Regarding osteoclastogenesis, inhibiting miRNA-21 in osteoblast was found to reduce the release of RANKL, therefore reducing osteoclastogenesis and leading to impaired bone remodeling (38). Finally, regarding angiogenesis, miRNA-21-5p targets Spry1 to promote vascular endothelial growth factor (VEGF) for angiogenesis (39).

From this previous evidence, miRNA-21 may have several roles in MRONJ development and healing. Finding the exact pathway that miRNA-21-5p is involved in with regard to MRONJ lesions could be beneficial for understanding the disease and future miRNA therapeutic strategies.

## Conclusions

The findings indicated that miRNA-21-5p expression levels were modified in the alveolar bones of the MRONJ rats. The expression level peaked 2 weeks post-tooth extraction. To our knowledge, no research has been conducted on the expression of miRNA-21-5p in the afflicted bone region. Additional research on the role of miRNA-21-5p could be beneficial as the result could be used to formulate promising miRNA therapies for patients who are administered bisphosphonates and have developed MRONJ.

## Data Availability

The datasets presented in this study can be found in online repositories. The names of the repository/repositories and accession number(s) can be found in the article/Supplementary Material.
