# Supplementary material for: MicroRNA-21-5p profile in the alveolar bone following tooth extraction in medication-related osteonecrosis of the jaw rat model
Source: Front Dent Med. 2024 Dec 17;5:1477274. doi: 10.3389/fdmed.2024.1477274 (PMC11797932; doi:10.3389/fdmed.2024.1477274)
Supplement: Supplementary file 1 [file Datasheet1.pdf]

Descriptives

	Day			Statistic	Std. Error
miR21exp	1Day	Mean		.4333	.21263
		95% Confidence Interval for Mean	Lower Bound	-.4815	
			Upper Bound	1.3482	
		5% Trimmed Mean		.	
		Median		.4900	
		Variance		.136	
		Std. Deviation		.36828	
		Minimum		.04	
		Maximum		.77	
		Range		.73	
		Interquartile Range		.	
		Skewness		-.676	1.225
		Kurtosis		.	.
	14Day	Mean		7.3067	2.06483
		95% Confidence Interval for Mean	Lower Bound	-1.5776	
			Upper Bound	16.1909	
		5% Trimmed Mean		.	
		Median		7.0600	
		Variance		12.791	
		Std. Deviation		3.57639	
		Minimum		3.86	
		Maximum		11.00	
		Range		7.14	
		Interquartile Range		.	

		Skewness		.309	1.225
		Kurtosis		.	.
	28day	Mean		1.8800	.53075
		95% Confidence Interval for Mean	Lower Bound	-.4037	
			Upper Bound	4.1637	
		5% Trimmed Mean		.	
		Median		1.4900	
		Variance		.845	
		Std. Deviation		.91929	
		Minimum		1.22	
		Maximum		2.93	
		Range		1.71	
		Interquartile Range		.	
		Skewness		1.565	1.225
		Kurtosis		.	.

Tests of Normality

		Kolmogorov-Smirnov[a]			Shapiro-Wilk		
	Day	Statistic	df	Sig.	Statistic	df	Sig.
miR21exp	1Day	.228	3	.	.982	3	.745
	14Day	.194	3	.	.996	3	.886
	28day	.331	3	.	.865	3	.281

a. Lilliefors Significance Correction

ANOVA

miR21exp

	Sum of Squares	df	Mean Square	F	Sig.
Between Groups	78.784	2	39.392	8.581	.017
Within Groups	27.543	6	4.590		
Total	106.327	8			

Multiple Comparisons

Dependent Variable: miR21exp

Tukey HSD

(I) Day	(J) Day	Mean Difference (I-J)	Std. Error	Sig.	95% Confidence Interval	
					Lower Bound	Upper Bound
1Day	14Day	-6.87333*	1.74937	.018	-12.2409	-1.5058
	28day	-1.44667	1.74937	.701	-6.8142	3.9209
14Day	1Day	6.87333*	1.74937	.018	1.5058	12.2409
	28day	5.42667*	1.74937	.048	.0591	10.7942
28day	1Day	1.44667	1.74937	.701	-3.9209	6.8142
	14Day	-5.42667*	1.74937	.048	-10.7942	-.0591

*. The mean difference is significant at the 0.05 level.
